# Time trends in pregnancy-related outcomes among women with type 1 diabetes mellitus, 2004–2017

**DOI:** 10.1038/s41372-020-0698-x

**Published:** 2020-06-02

**Authors:** Sarit Helman, Tamarra M. James-Todd, Zifan Wang, Andrea Bellavia, Jennifer A. Wyckoff, Shanti Serdy, Elizabeth Halprin, Karen O’Brien, Tamara Takoudes, Munish Gupta, Thomas F. McElrath, Florence M. Brown

**Affiliations:** 1 Harvard Medical School, Boston, MA USA; 2 Department of Environmental health, Harvard T.H. Chan School of Public Health, Boston, MA USA; 3 Department of Epidemiology, Harvard T.H. Chan School of Public Health, Boston, MA USA; 4 Department of Biostatistics, Harvard T.H. Chan School of Public Health, Boston, MA USA; 5 University of Michigan, Ann Arbor, MI USA; 6 Joslin Diabetes Center, Boston, MA USA; 7 Department of Obstetrics and Gynecology, Beth Israel Deaconess Hospital, Boston, MA USA; 8 Department of Neonatology, Beth Israel Deaconess Hospital, Boston, MA USA; 9 Division of Maternal Fetal Medicine, Brigham and Women’s Hospital, Boston, MA USA

**Keywords:** Type 1 diabetes, Epidemiology, Outcomes research, Health care economics

## Abstract

**Objective:**

To examine time trends in US pregnant women with type 1 diabetes mellitus for maternal characteristics and pregnancy outcomes.

**Study design:**

We abstracted clinical data from the medical records of 700 pregnant women from 2004 to 2017. For each time period, means and percentages were calculated. *P* values for trend were calculated using linear and logistic regression.

**Results:**

HbA1c in each trimester was unchanged across the analysis period. The prevalence of nephropathy decreased from 4.8% to 0% (*P* = 0.002). Excessive gestational weight gain increased (*P* = 0.01). Gestation length also increased (*P* = 0.01), as did vaginal deliveries (*P* = 0.03). There were no change in birthweight over time (*P* = 0.07) and the percentage of neonates with macrosomia and large for gestational age (LGA) neonates also remained unchanged.

**Conclusion:**

Obstetric guideline changes may have improved gestation length and mode of delivery; however, other outcomes need more attention, including excessive gestational weight gain, macrosomia, and LGA.

## Introduction

The prevalence of type 1 diabetes mellitus in pregnancy has almost doubled in the past few decades in the US [1]. Of concern, numerous studies show that maternal and fetal complications, such as perinatal mortality and fetal macrosomia, are substantially higher in women with type 1 diabetes than in women from the general population [2, 3]. In addition, pregnancies complicated by type 1 diabetes have a higher prevalence of cesarean section, preterm birth, and macrosomia and incur higher health care costs than pregnancies complicated by type 2 diabetes or gestational diabetes [4]. Optimization of glycemic control before and during pregnancy in women with type 1 diabetes is critical for improving pregnancy and delivery outcomes [5]. Interestingly, concurrent with the increase in type 1 diabetes prevalence, there have been rapid advances in technology for the purpose of improving glycemic control, including insulin pumps and continuous glucose monitors (CGM). However, it is unclear how widely technology to treat type 1 diabetes has been adopted for use in pregnancy or whether this has had any impact on pregnancy and delivery outcomes.

Several studies, conducted outside the US, have examined time trends in pregnancy-related outcomes among women with type 1 diabetes. Two Scandinavian studies which looked at the preconception period found increases in prepregnancy body mass index (BMI) over a 10–20 year time span [2, 6]; while other studies did not show increases in BMI during the same time period [7–9]. Preconception improvements in glycemic control were noted in one study, while others showed either deterioration or similar glycemic control levels [6, 10, 11]. Studies of trends in glycemic control during pregnancy demonstrated conflicting results with deterioration in a study from Finland but improvement in a study from the United Kingdom [6, 11]. Differences in these studies may be attributed to differences in population characteristics and health care delivery. Other European and Canadian studies have evaluated trends in delivery outcomes such as preterm births, cesarean deliveries, and birthweight finding increases in these outcomes over time. These studies also show that macrosomia remained higher than in the general population and did not change over time [6, 10, 12, 13]. While findings from these studies suggest the need for further evaluation, to our knowledge no study has specifically assessed time trends in pregnancy, treatment, and delivery outcomes among women with type 1 diabetes in a US population.

We aimed to examine time trends in prepregnancy maternal characteristics, treatment, and glucose control measures, along with pregnancy and delivery outcomes among US women with type 1 diabetes. With rapid advances in technology to treat type 1 diabetes, including the introduction of CGMs and the increasing use of insulin pumps, evaluating time trends for pregnancy and delivery outcomes could provide insights about this high-risk US population.

## Materials and methods

### Study population and eligibility

Clinical data were abstracted from the medical records of pregnant women with type 1 diabetes seeking care at the Joslin Diabetes Center and Beth Israel Deaconess Medical Center (BIDMC) Diabetes in Pregnancy Program. Most deliveries (663) were at BIDMC, however 37 deliveries occurred at other hospitals. Eligible women had clinically diagnosed type 1 diabetes, were 18 years or older with a singleton live birth pregnancy and delivered at BIDMC in the time period of 2004 through 2017. All women were managed by a multidisciplinary team that included endocrinologists, maternal-fetal medicine specialists, nurses, and dieticians. We included women who had multiple pregnancies as well. The study was approved by the Committee for Human Subjects at Joslin Diabetes Center.

### Prepregnancy maternal characteristics and treatment factors

We evaluated maternal prepregnancy characteristics, including age (continuous) and race (white or nonwhite). Prepregnancy weight and height were measured at the first prenatal clinic visit, prior to 14 weeks’ gestation. BMI was calculated as weight in kilograms per height in meters squared, and women were categorized as follows: underweight at <18.5 kg/m^2^, normal at 18.5–24.9 kg/m^2^, overweight at 25–29.9 kg/m^2^, and obese at ≥30 kg/m^2^ [14]. In addition, we abstracted data on the urine albumin–creatinine ratio (ACR) recorded up to 6 month prior to the last menstrual period or at the first prenatal visit. Normal ACR was defined as <30 mg/g. Microalbuminuria was defined as 30–299 mg/g and macroalbuminuria as ≥300 mg/g [15]. We based our analysis of insulin pump and CGM use initiated before pregnancy. Data on mode of glucose management were abstracted as either multiple daily injections or insulin pump use based on first prenatal visit. In addition, information on use of CGMs was taken from the medical records of the first prenatal visit.

### Pregnancy glycemic control and weight gain measures

Pregnancy glycemic control was evaluated using HbA1c values that were assessed in each trimester. Recommended levels of HbA1c were defined as <6.5% (48 mmol/mol) in the first trimester, and <6.0% (42 mmol/mol) for the second and third trimesters [16]. There was a change in glucose targets over the course of the study. From 2004 to 2008 glycemic targets were based on ADA’s 4th and 5th international workshops using the fasting and 2 h thresholds [17, 18]. From 2008 to 2017, targets were fasting/premeal, bedtime, overnight 60–99 and 1 h 100–129 based on ADA 2008 summary of evidence and consensus recommendations [19].

Gestational weight gain was calculated by subtracting the initial weight measured at the first prenatal visit from the weight measured in the third trimester. Excessive weight gain was assessed according to the revised recommendation of the Institute of Medicine (IOM), that account for maternal prepregnancy BMI [20]. The IOM guidelines were adopted at our institution in 2009. We applied the 2009 guidelines to the entire cohort to provide a consistent definition for the term “excessive weight gain” through the study period. Weight gain velocity was calculated by dividing the total weight gain by the gestational age at delivery.

### Delivery outcomes

Data on delivery mode, gestational age at birth, and birthweight were obtained from the medical records. We evaluated vaginal versus cesarean deliveries based on delivery records. Gestational age was based on last menstrual period and assessed as a continuous variable. In addition, preterm delivery was defined as <37 weeks gestation and early preterm birth was defined as <32 weeks gestation [21]. Birthweight was evaluated continuously, and birthweight percentiles were adjusted to account for differences in gestational age at delivery, infant sex, maternal height, weight, parity, and ethnicity [22]. Birthweight was evaluated categorically as well, as (1) macrosomia; or (2) large for gestational age (LGA). Macrosomia was defined as birthweight >4000 g. Infants with birthweight >90th percentile were classified as LGA. Those with birthweight below the 10th percentile were classified as small for gestational age (SGA). Infants with birthweight between the 10th and 90th percentile were considered as appropriate for gestational age.

We were able to extract time and date stamped neonatal glucose for the years 2008–2017 for infants who required stays in the neonatal intensive care unit (NICU). We discarded year 2008 as it represented a small and not well-defined portion of the 2004–2008 period. In addition, we excluded infants born in 2017 from the analysis due to a change in neonatal hypoglycemia thresholds from 40 to 45 mg/dL that year. Neonatal hypoglycemia was defined as < or =40 mg/dl.

### Statistical analysis

We calculated means and standard deviation for continuous variables and percentages for categorical variables. To calculate trends over time, the study cohort was divided into three groups: the first group included women who delivered in 2004–2008 and numbered 214 pregnancies; the second group delivered in 2009–2012 and numbered 215 pregnancies; and the third group delivered in 2013–2017 and numbered 271 women.

Linear regression was used, evaluating each time period as an indicator variable and assessing the pregnancy, treatment, and delivery outcomes as continuous variables. Logistic regression was used to evaluate the association between each time period and categorical outcome variables. All tests were two-tailed and *P* values for trend over time were calculated, with *P* < 0.05 as statistically significant time trend. Given that 188 women had more than one pregnancy within this dataset and thus had correlated data, we performed a sensitivity analysis that included only the first pregnancy within the dataset for each woman. For this analysis, a total of 512 women were included and all models were rerun within this restricted dataset.

In addition, to evaluate the adjusted associations between time period and maternal characteristics, pregnancy, treatment, and delivery outcomes, we constructed linear and logistic regression models. For continuous outcomes, using linear regression, we estimated the *β* coefficients with 95% confidence intervals (CIs). For binary outcomes, we used logistic regression to calculate odds ratios (ORs) with 95% CIs. For these analyses, we compared each of the later time periods with 2004–2008. We constructed crude models, as well as adjusted models, accounting for maternal age and BMI (Model 1) for maternal characteristics and treatment outcomes. For delivery and neonatal outcomes, we further adjusted for gestational weight gain (Model 2).

All analyses were performed using R version 3.3.2 (2016–10–31) and Stata/IC 15.1.

## Results

### Prepregnancy maternal characteristics and treatment factors

In the years from 2004 to 2017, there were 700 pregnancies that met the eligibility criteria in 512 women with type 1 diabetes. The prepregnancy maternal characteristics across the three evaluated time windows, together with *P* for trends, are presented in Table 1. During the study period, changes in mean maternal age and the prevalence of primiparous were negligible. The mean BMI and the prevalence of obesity remained the same, although there was a possible trend towards an increase in obesity over time. The prevalence of nephropathy decreased during the study period, with ACR >300 at 4.8% in 2004–2008 to 0% in 2013–2017 (*P* for trend =0.002). The use of insulin pumps increased from 50.0% in 2004–2008 to 58.1% in 2009–2012, and to 72.7% in 2013–2017 (*P* < 0.001). The use of CGMs also increased significantly with no use at the beginning of the study period to 2.2% in 2009–2012 and 39.9% in 2013–2017 (*P* < 0.001).

**Table 1:** Prepregnancy maternal characteristics in women with type 1 diabetes over 14 years of follow-up.

	Overall cohort^a^ *n* = 700	2004–2008 *n* = 214	2009–2012 *n* = 215	2013–2017 *n* = 271	*P* for trend
Age (years) (*n* = 679)	31.7 ± 5.2	31.9 ± 5.4	32.0 ± 5.5	31.3 ± 4.7	*P* = 0.19
White ethnicity (*n* = 700)	601 (85.8)	184 (85.9%)	182 (84.6%)	235 (86.7%)	*P* = 0.80
Primiparous (*n* = 695)	368 (52.9%)	112 (52.3%)	119 (56.4%)	137 (50.7%)	*P* = 0.67
BMI (kg/m^2^) (*n* = 577)	26.74 ± 5.3	26.45 ± 5.13	26.64 ± 4.99	26.99 ± 5.54	*P* = 0.31
Obesity^b^ (*n* = 577)	117 (20.2%)	26 (16.8%)	35 (20.6%)	56 (22.2%)	*P* = 0.19
Pump use (*n* = 652)	405 (62.1%)	83 (50.0%)	125 (58.1%)	197 (72.7%)	*P* < 0.001
CGM use (*n* = 652)	111 (17%)	0 (0%)	5 (2.2%)	106 (39.9%)	*P* < 0.001
Nephropathy^c^ (*n* = 597)	16 (3%)	9 (4.8%)	7 (4.1%)	0 (0%)	*P* = 0.002

Data are counts (percentages) or means ± SD. *P* values were calculated for trend.

*CGM* continuous glucose monitoring.

^a^Across all time periods. Analysis of 700 pregnancies in 512 women. Numbers may not sum up to 700 due to missing data on maternal characteristics.

^b^Obesity defined as BMI ≥ 30.

^c^Nephropathy defined as albumin–creatinine ratio ≥300.

### Pregnancy glycemic control and weight gain measures

In Table 2, we present the mean values of HbA1c during pregnancy. When analyzed continuously, there were no differences between the three groups for any trimester. In addition, we examined the percentage of women who reached the recommended levels of HbA1c in each trimester (<6.5% [48 mmol/mol] in the first trimester, and <6.0% [42 mmol/mol] for the second and third trimester), with a similar percentage of women reaching the recommendation across all time periods.

**Table 2:** Glucose control and gestational weight gain in women with type 1 diabetes during pregnancy over 14 years of follow-up.

	2004–2008 *n* = 214	2009–2012 *n* = 215	2013–2017 *n* = 271	*P* for trend
^a^HbA1c % 1st trimester (*n* = 636)	7.01 ± 1.31	6.88 ± 1.14	6.98 ± 1.05	*P* = 0.83
HbA1c mmol/mol 1st trimester (*n* = 636)	53 ± 9.89	52 ± 8.6	53 ± 8	*P* = 0.83
^b^HbA1c < 6.5% 1st trimester (*n* = 636)	72 (37.3%)	72 (37.9%)	84 (33.2%)	*P* = 0.35
^a^HbA1c % 2nd trimester (*n* = 660)	6.37 ± 0.94	6.24 ± 0.79	6.26 ± 0.74	*P* = 0.20
HbA1c mmol/mol 2nd trimester (*n* = 660)	46 ± 6.8	45 ± 6.3	45 ± 6	*P* = 0.20
^b^HbA1c < 6.0% 2nd trimester (*n* = 660)	70 (34.0%)	77 (38.5%)	87 (34.3%)	*P* = 1.00
^a^HbA1c % 3rd trimester (*n* = 648)	6.38 ± 0.79	6.31 ± 0.81	6.29 ± 0.68	*P* = 0.18
HbA1c mmol/mol 3rd trimester (*n* = 648)	46 ± 5.6	45 ± 5.8	45 ± 5	*P* = 0.18
^b^HbA1c < 6.0% 3rd trimester (*n* = 648)	60 (29.6%)	59 (31.1%)	76 (29.8%)	*P* = 0.97
Gestational weight gain (lbs.) (*n* = 585)	20.38 ± 10.33	19.11 ± 9.34	21.31 ± 10.91	*P* = 0.28
Excessive weight gain during pregnancy (*n* = 527)	23, 16.3%	23, 15.4%	62, 26.2%	*P* = 0.01

Data are means ± SD or *n* (%). *P* values were calculated for trend.

^a^Maternal HbA1c data were available for *N* = 636 in the first trimester, *N* = 660 in the second trimester, and *N* = 648 in the third trimester.

^b^Target HbA1c value were <6.5% (48 mmol/mol) in the first trimester and <6.0% (42 mmol/mol) in the second and third trimester. Numbers may not sum up to 700 due to missing data on glucose control and weight gain measures.

Overall, mean gestational weight gain remained unchanged across each of the time periods (Table 2). However, when assessed categorically based on IOM guidelines that account for prepregnancy BMI, the percentage of women with excessive gestational weight gain increased significantly with 16.3% gaining excessive weight in 2004–2008 and 26.2% in 2013–2017 (*P* = 0.02). When evaluating weight gain velocity, no significant differences were seen over time.

### Delivery outcomes

Table 3 presents delivery outcomes. The average gestational age at delivery lengthened from 37.0 weeks in 2004–2008 to 37.1 weeks in 2009–2012 and 37.4 weeks in 2013–2017 (*P* = 0.03). However, preterm birth did not change significantly over time for either measure (gestational age <37 weeks or <32 weeks). There was an insignificant tendency towards increased birthweight but birthweight percentiles did not change. The prevalence of macrosomia and LGA remained high but did not change significantly over time. The prevalence of SGA remained low and did not change over time. Vaginal deliveries increased from 21.1% in 2004–2008 to 24.2% in 2009–2012, and 29.6% in 2013–2017 (*P* = 0.03). The prevalence of primary cesarean deliveries tended to decrease over time and the direction was similar to the overall reduction in cesarean deliveries.

**Table 3:** Delivery outcomes in women with type 1 diabetes over 14 years of follow-up.

	2004–2008 *n* = 214	2009–2012 *n* = 215	2013–2017 *n* = 271	*P* for trend
Birthweight (kg) (*n* = 696)	3.63 ± 0.74	3.62 ± 0.73	3.75 ± 0.78	*P* = 0.07
Birthweight percentiles (*n* = 696)	82.0 ± 24.9	79.4 ± 25.8	82.4% ± 25.1	*P* = 0.77
Macrosomia (*n* = 696)	67 (31.5%)	65 (30.2%)	100 (37.2%)	*P* = 0.16
LGA (*n* = 696)	121 (56.8%)	113 (52.8%)	166 (61.7%)	*P* = 0.24
SGA (*n* = 696)	7 (3.3%)	6 (2.8%)	5 (1.9%)	*P* = 0.32
Gestational age at delivery (weeks) (*n* = 698)	37.0 ± 2	37.1 ± 2	37.4 ± 2	*P* = 0.01
Preterm deliveries before 37 weeks (*n* = 698)	61 (28.6%)	54 (25.2%)	63 (23.2%)	*P* = 0.18
Preterm deliveries before 32 weeks (*n* = 698)	4 (1.9%)	3 (1.4%)	4 (1.5%)	*P* = 0.74
Vaginal deliveries (*n* = 698)	45 (21.1%)	52 (24.2%)	80 (29.6%)	*P* = 0.03
Primary cesarean delivery (*n* = 368)^a^	91 (81.3%)	86 (72.3%)	97 (70.8%)	*P* = 0.06
Neonatal hypoglycemia that required NICU (*n* = 422, during 2009–2016)^b^	–	34 (16.0%)	58 (27.8%)	*P* = 0.004

Data are counts (percentages) or means ± SD. *P* values were calculated for trend. Analysis of 700 pregnancies in 512 women. Numbers may not sum up to 700 due to missing data on delivery outcomes.

*LGA* large for gestational age, *SGA* small for gestational age.

^a^Primary cesarean deliveries (percentages) were restricted to 368 primiparous women. The number of primiparous women during 2004–2008, 2009–2012, and 2013–2017 were 112, 119, and 137, respectively.

^b^We were able to extract neonatal glucose for the years 2008–2017 for infants who required stays in the neonatal intensive care unit (NICU). We discarded year 2008 as it only represents a small and not well-defined portion of the 2004–2008 period. In addition, we excluded infants born in 2017 from the analysis due to a change neonatal hypoglycemia thresholds from 40 towards 45 mg/dL that year. Of the 422 neonates (213 during 2009–2012, 209 during 2013–2016) who were born at BIDMC during 2009–2016, 170 (40%) were treated in the NICU. 92 (22%) were treated in the NICU and had one or more blood glucose levels ≤40 mg/dL.

Of the 422 neonates (213 during 2009–2012, 209 during 2013–2016) who were born at BIDMC during 2009–2016, 170 (40%) were treated in the NICU. Ninety-two (22%) were treated in the NICU and had one or more blood glucose levels ≤40 mg/dL. Neonatal hypoglycemia occurred more frequently in the 2013–2016 period (Table 3).

### Sensitivity analysis

The results of the sensitivity analysis are presented in Tables S1–S3 in the Supplementary materials. This analysis included only the first pregnancy for each woman in the dataset. As expected, maternal age was younger and decreased in the sensitivity analysis subgroups especially in the 2009–2012 and 2013–2017 (*P* = 0.005), and the prevalence of primiparous increased significantly (*P* < 0.001). The increase in the use of insulin pumps and CGMs and the decrease in the prevalence of nephropathy remained significant. Glucose control results were similar to the full dataset, with no difference between the subgroups during pregnancy and the increase in excessive weight gain during pregnancy remained significant as well (*P* = 0.03). Finally, we found similar results for no change in birthweight, prevalence of macrosomia, LGA and SGA and preterm deliveries. We found weaker *P* values for the increase in vaginal deliveries and gestational age at delivery, possibly due to limited power attributed to smaller sample size.

### Adjusted associations between time period and outcomes

Adjusted associations between time period and maternal characteristics, pregnancy and delivery outcomes are presented in Supplementary Tables S4–S6. Comparing each of the later time periods with 2004–2008, we found an ~65% increase in excessive GWG when comparing 2013–2017 with 2004–2008, after adjustment for age and BMI (adj. OR: 1.65; 95% CI: 0.96–2.83). We also found an almost threefold increase in pump use in 2013–2017 compared with 2004–2008 and a 31-fold increase in CGM 2013–2017 compared with 2009–2012 (adj. OR for pump use: 2.85; 95% CI: 1.79, 4.56 and adj. OR for CGM use: 31.4; 95% CI: 11.2, 88.4, respectively). There was also significantly greater gestational age and a higher odds of vaginal deliveries in the later years compared with earlier years. Borderline findings included a 40% increase in LGA 2013–2017 compared with 2004–2008 and a 30% increase in macrosomia.

## Discussion

Our study sought to focus on quantifying maternal variables and selected delivery outcomes, such as maternal BMI, gestational weight gain and birthweight, that have been possibly more intractable over time despite technological advances in diabetes management. We found significant time trends in several prepregnancy maternal characteristics, treatment measures, and pregnancy and delivery outcomes. First, while prepregnancy anthropometry factors remained the same, nephropathy decreased. Second, the percentage of women who use insulin pumps and CGMs increased dramatically between 2004 and 2017. There were no changes in glycemic control based on mean trimester-specific HbA1c levels across pregnancy. Third, the percentage of women with type 1 diabetes, who gained excessive gestational weight increased significantly. Fourth, gestational age at delivery increased and the percentage of cesarean deliveries decreased. However, birthweight percentile, the prevalence of preterm birth, as well as LGA and fetal macrosomia did not change over the study period, although adjusted models demonstrated nonsignificant directional trends towards more LGA and macrosomia. We also found increased neonatal hypoglycemia.

During the study period, there were significant changes in glucose management that include an increase in the use of insulin pumps and CGMs. Previous studies examining the efficacy of these techniques showed conflicting results [23–27]. We did not demonstrate any changes in mean HbA1C levels before and during pregnancy and the percentage of women with higher than recommended levels of HbA1c during pregnancy remained fairly high at 60–70%. This inconsistent attainment of HbA1c targets is worrisome since poor glycemic control is known to increase adverse perinatal outcomes [28, 29]. It has been observed that HbA1c is too blunt a tool to detect important improvements in glucose in pregnancy and that CGM analysis is far better at giving this information in pregnancy [30]. As noted in the CONCEPTT trial, HbA1c may not fully reflect glycemic control, including time spent in target range [24]. Possible explanations for our results could be that the users of insulin pumps and CGMs might have had more severe forms of diabetes to begin with or that self-adjustment of insulin dosing may be more challenging during pregnancy [26]. A recent study demonstrated that the prevalence of LGA is higher in insulin pump users than those who use multiple daily injections and this may be mediated by excess weight gain in pregnancy [31]. Moreover, it is possible that the lack of improvement in glycemic control, as reflected by HbA1c, might be due to a false sense of reassurance for individual patients using these newer technologies, with less concern about counting carbohydrates and pre-bolusing before meals.

Despite the fact that we did not find a significant change over time for trimester-specific HbA1c, the prevalence of nephropathy decreased significantly. In pregnancy, diabetic nephropathy is associated with several adverse outcomes in pregnancy including hypertensive disorders, preterm deliveries, intrauterine growth restriction, and stillbirth [28]. Of interest, within this population this trend may have started prior to the start of the present study, as earlier studies in late 1970s and 1980s at the Joslin Diabetes Center reported nephropathy prevalence of 9.6% and 14% compared with our 0% for the 2013–2017 time period [32, 33]. This decrease in the prevalence of diabetic nephropathy may indicate improvements in glycemic control that preceded the start of the present study’s time period or the increasing use of renin angiotensin system blocking medications in our population with microalbuminuria, in the years leading up to pregnancy.

A prepregnancy factor that complicates some pregnancies is obesity, as it is an important risk factor for perinatal complications [2, 34]. We did not find significant change in maternal BMI, although there was a suggestive increase. However, we did find a significant increase in the percentage of women with gestational weight gain that exceeded the IOM recommendations, based on their prepregnancy BMI. The IOM guidelines were adopted at our institution in 2009. We applied the 2009 guidelines to the entire cohort to provide a consistent definition for the term “excessive weight gain” through the study period. We acknowledge that the changes in the 2009 IOM guidelines compared with the 1990 guidelines, especially the determination of discrete BMI categories and the establishment of upper ranges for weight gain in the obese category may have had an impact on of reducing excessive weight gain. Yet despite this improved focus on reducing excessive weight gain, we saw an increase in excessive weight gain over the years of our study.

Excessive gestational weight gain is associated with several adverse outcomes such as hypertensive disorders, cesarean delivery, and macrosomia, which are complications that are more prevalent in women with type 1 diabetes [35, 36]. The increase in the prevalence of women with excessive gestational weight gain by BMI category could be attributed to the possible tendency towards increasing prevalence of prepregnancy obesity in this population, with gestational weight gain categories relying on prepregnancy BMI to determine excessive, adequate, or inadequate weight gain during pregnancy. Specifically, the prevalence of obesity in this study population increased from 16.8% in 2004–2008 to 22.2% in 2013–2017. In fact, most of the women who had excessive gestational weight gain were categorized as obese, with only 18% of women with normal BMI gaining excessive weight based on IOM guidelines compared with 72% of obese women. It is also possible that dietary intake patterns may have changed to contribute to more women exceeding the weight gain targets. We do not have dietary information on our cohort but a recent subgroup analysis from the CONCEPTT Study demonstrated that close to 50% of daily carbohydrate consumed by their study population were derived from non-recommended sources such as sugars, preserves, confectionery sources, biscuits, and cakes [37]. Thus, it may be important to consider strategies for communicating appropriate weight gain for a somewhat increasingly obese population of women with type 1 diabetes.

During the study period, there were several changes in obstetrical practice, including a change in the approach for the timing for induction of labor for women with type 1 diabetes. Traditionally, the goal of induction of labor for women with type 1 diabetes was the prevention of stillbirth and macrosomia along with the complications associated with these conditions [38]. According to current recommendation made by the American College of Obstetrics and Gynecology delivery is no longer induced prior to 39 weeks gestation unless glucose control is suboptimal, or maternal or fetal complications accrue [5, 39]. This change could have influenced our study results towards longer gestational age at delivery and higher birthweights in the latter time periods. In fact, a randomized control trial conducted two decades ago corroborates these findings, with women having higher birthweight babies in the expectant management group compared with the induction of labor group [40]. We found a decrease in cesarean deliveries in the present study, as opposed to other studies that examined trends in pregnancy outcomes in women with type 1 diabetes [9, 12]. Guidelines regarding labor management have changed in recent years as a concerted effort is being made to reduce the cesarean section rate in all obstetric populations. The definition of labor dystocia has been revisited with more generous definitions of arrested labor, fetal heart rate interpretation has been standardized, and breech versions and vaginal births after cesarean are offered more frequently. All of these measures are employed more uniformly with the intention of decreasing the cesarean section rate nationwide [41].

Likewise, there were changes in cutoffs for hypoglycemia. It is possible that these changes may have led to the identification of more neonatal hypoglycemia. However, excessive gestational weight gain is positively associated with neonatal hypoglycemia [42, 43]. As such, it cannot be ruled out that the higher prevalence of excessive gestational weight gain over time could be contributing to a higher prevalence of neonatal hypoglycemia. More work is needed to further understand these trends and their associations in a population of pregnant women with type 1 diabetes.

We found no substantial differences between our main analysis and the sensitivity analysis, indicating the validity of the main analysis.

This study has several limitations. First, this study was conducted in a single site, and therefore may not be generalizable to other study populations. However, the Joslin Diabetes Center has a large catchment area and is located in Massachusetts where in 2006 a law mandated that residents obtain a minimum level of insurance coverage which could be free or subsidized based on earnings, allowing for improved health care access [44]. Second, this dataset was not created for research purposes and consequently, we did not have reliable data on the duration of diabetes. Percentage of missing data is shown in the appendix. We acknowledge that there is missing data for BMI and gestational weight gain. The missing data is noted more often in the earlier years of this study when height and weight were not as rigorously recorded as they are now. In addition, some 1st trimester missing data (HbA1c, weight) were due to presenting after the first trimester of pregnancy. However, we found no differences in birthweight, birthweight percentiles, and gestational age at delivery in pregnancies missing BMI compared with not missing BMI data. Further, 82–99% of our data were complete and we were able to evaluate a variety of prepregnancy, pregnancy, treatment, and delivery-related outcomes across more than a decade within a single clinic. Fourth, we recorded CGM use based on the use at the beginning of pregnancy. However, CGMs may not have been continuously used and worn throughout pregnancy. The effectiveness of CGM in pregnancies with type 1 diabetes appears to come from continuous wear and usage in pregnancy, rather than intermittent usage. Therefore CGM usage based on whether a woman wore a CGM at the beginning of pregnancy may not mean that it was used consistently throughout pregnancy, with implications for its effectiveness. Finally, the presence of anemia could possibly impact HbA1c levels in this pregnant population. We did not include hemoglobin levels as a part of this study; while clinically used to evaluate glycemic control, results of HbA1c should be interpreted with caution [45].

Despite these limitations, this study has several strengths. The main strength of our study is that it is one of the largest datasets of women with type 1 diabetes in the US. Furthermore, the study evaluates more than a decade of data allowing detection of time trends in a high-risk population. The data includes information on maternal prepregnancy characteristics, treatment and glycemic control measures during pregnancy, and pregnancy and delivery outcomes. Finally, this is the first study to examine time trends in pregnant women with type 1 diabetes living in the US.

In conclusion, over the last ~15 years, rapid technological changes have occurred with a significant increase in the use of insulin pumps and CGM. In addition, changes in obstetrical guidelines also occurred. With this, certain pregnancy and delivery-related outcomes improved, including increased gestational age and decreased cesarean delivery. However, other pregnancy-related outcomes have been adversely impacted, including increases in the prevalence of excessive gestational weight gain and no improvements in infant birthweight measures. As such, this study may provide some insight into adverse outcomes that may need to have focused attention for improving pregnancy health in women with type 1 diabetes. Future studies will be aimed at evaluating associations between maternal characteristics, treatment modalities and glycemic control with delivery outcomes in this population.

## Supplementary information

supplemental tables 1–3

supplemental tables 4–6

Appendix
