# Supplementary material for: Time trends in pregnancy-related outcomes among women with type 1 diabetes mellitus, 2004–2017
Source: J Perinatol. 2020 Jun 2;40(8):1145–53. doi: 10.1038/s41372-020-0698-x (PMC7375951; doi:10.1038/s41372-020-0698-x)
Supplement: Supplementary file 1 — supplemental tables 1–3 [file 41372_2020_698_MOESM1_ESM.docx]

**Supplementary materials**

**Table S1: Pre-pregnancy maternal characteristics in women with type 1 diabetes over 14 years of follow-up among first pregnancy in dataset (sensitivity analysis)**

|  | **Total Cohort**  **n=512** | **2004-2008**  **n=186** | **2009-2012**  **n=152** | **2013-2017**  **n=174** | **p for trend** |
| --- | --- | --- | --- | --- | --- |
| **Age (years) (n=497)** | 30.9±5.3 | 31.7±5.6 | 31.1±5.6 | 30.1±4.7 | p=0.005 |
| **White Ethnicity (n=512)** | 433 (84.5%) | 159(85.4%) | 128 (84.2%) | 146 (83.9%) | p=0.90 |
| **Primiparous (n=510)** | 368 (72%) | 112(60.2%) | 119 (78.8%) | 137 (79.2%) | p<0.001 |
| **BMI (kg/m2) (n=419)** | 26.6±5.3 | 26.43±5.32 | 26.62±5.17 | 26.7.8±5.47 | p=0.56 |
| ***Obesity (n=419)** | 79 (18.8%) | 23 (16.9%) | 23 (18.9%) | 33 (20.5%) | p=0.43 |
| **Pump use (n=464)** | 267 (52.1%) | 67 (48.6%) | 85 (55.9%) | 115 (66.1%) | p=0.002 |
| **†CGM use (n=464)** | 70 (15%) | 0 (0%) | 4 (2.6%) | 66 (37.9%) | p<0.001 |
| **‡Nephropathy (n=425)** | 13 (3%) | 7 (4.4%) | 6 (5.1%) | 0 (0%) | p=0.03 |

Data are counts (percentages) or means ± SD. P values were calculated for trend. *Obesity defined as BMI≥30, †CGM= continuous glucose monitoring, ‡Nephropathy defined as albumin creatinine ratio ≥300. Analysis of 512 women.

|  | 2004-2008  n=186 | 2009-2012  n=152 | 2013-2017  n=174 | p for trend |
| --- | --- | --- | --- | --- |
| **HbA1c % 1st trimester (n=457)** | 7.02 ± 1.31 | 6.86 ± 1.21 | 7.02 ± 1.18 | p=0.98 |
| **HbA1c<6.5% 1st trimester (n=457)** | 61 (36.7%) | 54 (40.9%) | 56(35.2%) | p=0.79 |
| **HbA1c % 2nd trimester (n=483)** | 6.38± 0.99 | 6.25 ± 0.89 | 6.28 ± 0.81 | p=0.31 |
| **HbA1c<6.0% 2nd trimester (n=483)** | 64 (35.8%) | 60 (42%) | 53 (32.9%) | p=0.62 |
| **HbA1c % 3rd trimester (n=475)** | 6.36 ± 0.78 | 6.25 ± 0.71 | 6.25 ± 0.74 | p=0.21 |
| **HbA1c<6.0% 3rd trimester (n=475)** | 55 (31.1%) | 47 (35.1%) | 57 (34.8%) | p=0.47 |
| **Gestational weight gain (lbs.) (n=430)** | 20.4 ± 10.4 | 19.6 ± 9.9 | 21.065 ± 12 | p=0.57 |
| **Excessive weight gain during pregnancy (n=381)** | 20(16.1%) | 20(18.7%) | 41 (27.3%) | p=0.03 |

**Table S2: Glucose control and weight gain during pregnancy in women with type 1 diabetes over 14 years of follow-up among first pregnancy in dataset (sensitivity analysis)**

Data are means ± SD or n (%). p values were calculated for trend. ‡ Maternal HbA1c data were available for N = 457 in the first trimester, N = 483 in the second trimester, and N = 475 in the third trimester. † Target HbA1c value were <6.5% in the first trimester and <6.0% in the second and third trimester. Numbers may not sum up to 512 due to missing data on glucose control and weight gain measures

**Table S3: Delivery outcomes in women with type 1 diabetes over 14 years of follow-up among first pregnancy in dataset (sensitivity analysis)**

|  | **2004-2008**  **n=186** | **2009-2012**  **n=152** | **2013-2017**  **n=174** | **p for trend** |
| --- | --- | --- | --- | --- |
| **Birth weight (kg) (n=509)** | 3.58 ± 0.75 | 3.57 ± 0.70 | 3.60± 0.78 | p=0.74 |
| **Macrosomia (n=509)** | 55 (29.7%) | 41 (27.0%) | 49 (28.5%) | p=0.79 |
| ***LGA (n=509)** | 105 (56.6%) | 77(51.0%) | 107 (62.2%) | p=0.29 |
| **†SGA (n=509)** | 7 (3.8%) | 2(1.3%) | 5 (2.9%) | P=0.60 |
| **Gestational age at delivery (weeks) (n=510)** | 36.6 ± 2 | 37.2 ± 2 | 37.3 ± 2 | p=0.05 |
| **Preterm deliveries before 37 weeks (n=510)** | 55(29.7%) | 39(25.8%) | 44 (25.3%) | p=0.34 |
| **Preterm deliveries before 32 weeks (n=510)** | 4 (2.2%) | 2 (1.3%) | 4 (2.3%) | p=0.93 |
| **Vaginal deliveries (n=510)** | 40 (21.6%) | 40 (26.3%) | 52 (30.1%) | p=0.07 |
| **Neonatal hypoglycemia that required NICU (n=330, during 2009-2016)** | / | 32 (16.8%) | 38 (27.3%) | p=0.02 |

Data are counts (percentages) or means ± SD. P values were calculated for trend. *LGA= large for gestational age, †SGA= small for gestational age
