# Supplementary material for: Time trends in pregnancy-related outcomes among women with type 1 diabetes mellitus, 2004–2017
Source: J Perinatol. 2020 Jun 2;40(8):1145–53. doi: 10.1038/s41372-020-0698-x (PMC7375951; doi:10.1038/s41372-020-0698-x)
Supplement: Supplementary file 2 — supplemental tables 4–6 [file 41372_2020_698_MOESM2_ESM.docx]

**Supplemental Table S4.** Pre-pregnancy maternal characteristics in women with type 1 diabetes over 14 years of follow-up, comparing 2009-2012 and 2013-2017 to 2004-2008.

|  | **Crude model**  **β or OR (95% CI)** | | | | **Adjusted model 1 ^a^**  **β or OR (95% CI)** | | | |
| --- | --- | --- | --- | --- | --- | --- | --- | --- |
|  | **2004-2008** | **2009-2012** | **2013-2017** | **p-trend** | **2004-2008** | **2009-2012** | **2013-2017** | **p-trend** |
| **Age (years) (n=679)** | REF | β = 0.18  (-0.82, 1.17) | β = -0.60  (-1.53, 0.32) | 0.19 | / | / | / | / |
| **White Ethnicity (n=700)** | REF | OR = 0.90  (0.53, 1.54) | OR = 1.06  (-0.63, 1.79) | 0.80 | / | / | / | / |
| **Primiparous (n=695)** | REF | OR = 1.18  (0.80, 1.73) | OR = 0.94  (0.66, 1.34) | 0.67 | REF | OR = 1.22  (0.77, 1.95) | OR = 0.78  (0.51, 1.19) | 0.17 |
| **BMI (kg/m^2^) (n=577)** | REF | β = 0.19  (-0.96, 1.33) | β = 0.53  (-0.52, 1.59) | 0.31 | / | / | / | / |
| ***Obesity**  **(n=577)** | REF | OR = 1.29  (0.73, 2.26) | OR = 1.42  (0.85, 2.37) | 0.19 | REF | OR = 1.22 ^b^  (0.69, 2.16) | OR = 1.42 ^b^  (0.85, 2.38) | 0.18 ^b^ |
| **Pump use (n=652)** | REF | OR = 1.39  (0.92, 2.09) | OR = 2.66  (1.78, 3.99) | <0.001 | REF | OR = 1.50  (0.92, 2.43) | OR = 2.85  (1.79, 4.56) | <0.001 |
| **†CGM use (n=652)** | / | REF ^c^ | OR = 27.0  (10.8, 67.7) | <0.001 | / | REF ^c^ | OR = 31.4  (11.2, 88.4) | <0.001 |
| **‡Nephropathy (n=597)** | REF | OR = 0.85  (0.31, 2.33) | OR = 0 | <0.01 | REF | OR = 1.0  (0.31, 3.19) | OR = 0 | 0.01 |

Analysis of 700 pregnancies in 512 women. Numbers may not sum up to 700 due to missing data on maternal characteristics.

Year 2004-2008 was used as the reference group for all analyses, except for CGM use (since CGM was not used during that period).

β coefficients and 95% CIs were obtained for continuous outcome variables from linear regression models, and odds ratios (ORs) and 95% CIs were obtained for binary outcome variables from logistic regression models. P-values were calculated for trend.

*Obesity defined as BMI≥30. †CGM: continuous glucose monitoring. ‡Nephropathy defined as albumin creatinine ratio ≥300.

^a^ Adjusted model 1: adjusted for age (years) and BMI (kg/m^2^). “ / ”: adjustment not applicable.

^b^ Only adjusted for age (years).

^c^ 2009-2012 was used as the reference period for CGM use, since CGM was not used during 2004-2008.

**Supplemental Table S5.** Glucose control and gestational weight gain in women with type 1 diabetes over 14 years of follow-up, comparing 2009-2012 and 2013-2017 to 2004-2008.

|  | **Crude model**  **β or OR (95% CI)** | | | | **Adjusted Model 1 ^a^**  **β or OR (95% CI)** | | | |
| --- | --- | --- | --- | --- | --- | --- | --- | --- |
|  | **2004-2008** | **2009-2012** | **2013-2017** | **p-trend** | **2004-2008** | **2009-2012** | **2013-2017** | **p-trend** |
| **HbA1c % 1st trimester**  **(n=636)** | REF | β = -0.14  (-0.37, 0.09) | β = -0.03  (-0.25, 0.18) | 0.83 | REF | β = -0.03  (-0.27, 0.20) | β = 0.10  (-0.12, 0.31) | 0.30 |
| **HbA1c mmol/mol 1st trimester**  **(n=636)** | REF | β = -1.49  (-4.01, 1.03) | β = -0.37  (-2.73, 1.99) | 0.83 | REF | β = -0.37  (-2.91, 2.16) | β = 1.08  (-1.27, 3.41) | 0.30 |
| **HbA1c<6.5% 1st trimester**  **(n=636)** | REF | OR = 1.03  (0.68, 1.55) | OR = 0.84  (0.56, 1.24) | 0.35 | REF | OR = 0.97  (0.61, 1.54) | OR = 0.72  (0.47, 1.11) | 0.11 |
| **HbA1c % 2nd trimester**  **(n=660)** | REF | β = -0.13  (-0.29, 0.03) | β = -0.10  (-0.26, 0.05) | 0.20 | REF | β = -0.07  (-0.23, 0.08) | β = -0.01  (-0.16, 0.13) | 0.96 |
| **HbA1c mmol/mol 2nd trimester**  **(n=660)** | REF | β = -1.42  (-3.16, 0.33) | β = -1.14  (-2.79, 0.50) | 0.20 | REF | β = -0.80  (-2.52, 0.93) | β = -0.14  (-1.73, 1.45) | 0.96 |
| **HbA1c<6.0% 2nd trimester**  **(n=660)** | REF | OR = 1.22  (0.81, 1.82) | OR = 1.01  (0.69, 1.49) | 1.00 | REF | OR = 1.16  (0.74, 1.82) | OR = 0.87  (0.57, 1.33) | 0.43 |
| **HbA1c % 3rd trimester**  **(n=648)** | REF | β = -0.07  (-0.22, 0.08) | β = -0.10  (-0.24, 0.04) | 0.18 | REF | β = -0.11  (-0.27, 0.05) | β = -0.07  (-0.21, 0.07) | 0.42 |
| **HbA1c mmol/mol 3rd trimester**  **(n=648)** | REF | β = -0.79  (-2.42, 0.84) | β = -1.05  (-2.57, 0.47) | 0.18 | REF | β = -1.19  (-2.90, 0.52) | β = -0.75  (-2.30, 0.81) | 0.42 |
| **HbA1c<6.0% 3rd trimester**  **(n=648)** | REF | OR = 1.07  (0.70, 1.65) | OR = 1.01  (0.68, 1.52) | 0.97 | REF | OR = 1.05  (0.65, 1.70) | OR = 0.86  (0.55, 1.34) | 0.46 |
| **Gestational weight gain (lbs.)**  **(n=585)** | REF | β = -1.26  (-3.43, 0.91) | β = 0.94  (-1.03, 2.91) | 0.28 | REF | β = -0.39  (-2.54, 1.77) | β = -1.33  (-0.62, 3.28) | 0.13 |
| **Excessive weight gain during pregnancy**  **(n=527)** | REF | OR = 0.92  (0.49, 1.73) | OR = 1.80  (1.06, 3.07) | 0.01 | REF | OR = 0.93 ^b^  (0.49, 1.76) | OR = 1.65 ^b^  (0.96, 2.83) | 0.04 ^b^ |

Analysis of 700 pregnancies in 512 women. Numbers may not sum up to 700 due to missing data on maternal characteristics.

Year 2004-2008 was used as the reference group for all analyses.

β coefficients and 95% CIs were obtained for continuous outcome variables from linear regression models, and odds ratios (ORs) and 95% CIs were obtained for binary outcome variables from logistic regression models. P-values were calculated for trend.

^a^ Adjusted model 1: adjusted for age (years) and BMI (kg/m^2^).

^b^ Only adjusted for age (years), as BMI was already incorporated in the definition of excessive weight gain during pregnancy.

**Supplemental Table S6.** Delivery and neonatal outcomes in women with type 1 diabetes over 14 years of follow-up, comparing 2009-2012 and 2013-2017 to 2004-2008.

|  | **Crude model**  **β or OR (95% CI)** | | | | **Adjusted Model 1 ^a^**  **β or OR (95% CI)** | | | | **Adjusted Model 2 ^b^**  **β or OR (95% CI)** | | | |
| --- | --- | --- | --- | --- | --- | --- | --- | --- | --- | --- | --- | --- |
|  | **2004-2008** | **2009-2012** | **2013-2017** | **p-trend** | **2004-2008** | **2009-2012** | **2013-2017** | **p-trend** | **2004-2008** | **2009-2012** | **2013-2017** | **p-trend** |
| **Birthweight (kg)**  **(n=697)** | REF | β = -0.02  (-0.16, 0.13) | β = 0.12  (-0.02, 0.25) | 0.07 | REF | β = -0.00  (-0.16, 0.16) | β = 0.15  (0.00, 0.30) | 0.03 | REF | β = -0.00  (-0.17, 0.16) | β = 0.14  (-0.01, 0.29) | 0.05 |
| **Birthweight percentiles**  **(n=697)** | REF | β = -2.60  (-7.39, 2.19) | β = 0.46  (-4.08, 5.00) | 0.77 | REF | β = -3.21  (-8.70, 2.27) | β = 1.21  (-3.84, 6.26) | 0.50 | REF | β = -3.24  (-8.88, 2.40) | β = 1.60  (-3.52, 6.72) | 0.41 |
| **Macrosomia**  **(n=697)** | REF | OR = 0.94  (0.63, 1.42) | OR = 1.29  (0.88, 1.89) | 0.16 | REF | OR = 0.98  (0.61, 1.58) | OR = 1.34  (0.87, 2.06) | 0.15 | REF | OR = 0.96  (0.57, 1.60) | OR = 1.31  (0.83, 2.07) | 0.20 |
| **LGA**  **(n=696)** | REF | OR = 0.85  (0.58, 1.25) | OR =1.23  (0.85, 1.77) | 0.24 | REF | OR = 0.86  (0.55, 1.34) | OR = 1.29  (0.86, 1.95) | 0.16 | REF | OR = 0.89  (0.56, 1.41) | OR = 1.39  (0.91, 2.14) | 0.09 |
| **SGA**  **(n=696)** | REF | OR = 0.85  (0.28, 2.57) | OR = 0.56  (0.17, 1.78) | 0.32 | REF | OR = 1.18  (0.20, 4.56) | OR = 0.54  (0.13, 2.25) | 0.36 | REF | OR = 1.27  (0.28, 5.88) | OR = 0.53  (0.10, 2.67) | 0.40 |
| **Gestational age at delivery (weeks)**  **(n=698)** | REF | β = 0.21  (-0.16, 0.59) | β = 0.47  (0.11, 0.82) | 0.01 | REF | β = 0.33  (-0.10, 0.75) | β = 0.51  (0.12, 0.91) | 0.01 | REF | β = 0.26  (-0.18, 0.71) | β = 0.43  (0.03, 0.84) | 0.04 |
| **Preterm deliveries before 37 weeks**  **(n=698)** | REF | OR = 0.84  (0.55, 1.29) | OR = 0.76  (0.50, 1.14) | 0.18 | REF | OR = 0.73  (0.44, 1.20) | OR = 0.68  (0.43, 1.08) | 0.11 | REF | OR = 0.74  (0.43, 1.25) | OR = 0.69  (0.43, 1.12) | 0.15 |
| **Preterm deliveries before 32 weeks**  **(n=698)** | REF | OR = 0.74  (0.16, 3.36) | OR = 0.78  (0.19, 3.17) | 0.74 | REF | OR = 0.31  (0.03, 3.04) | OR = 0.81  (0.18, 3.70) | 0.87 | REF | OR = 0.52  (0.05, 6.07) | OR = 1.07  (0.18, 6.34) | 0.88 |
| **Vaginal deliveries**  **(n=698)** | REF | OR = 1.19  (0.76, 1.87) | OR = 1.57  (1.03, 2.39) | 0.03 | REF | OR = 1.14  (0.67, 1.93) | OR = 1.60  (1.00, 2.58) | 0.04 | REF | OR = 1.14  (0.64, 2.01) | OR = 1.79  (1.08, 2.96) | 0.02 |
| **Neonatal hypoglycemia that required NICU**  **(n=422, during 2009-2016) ^c^** | / | REF | OR = 2.02  (1.26, 3.25) | 0.004 | / | REF | OR = 2.63  (1.52, 4.55) | <0.001 | / | REF | OR = 2.90  (1.58, 5.30) | <0.001 |

Analysis of 700 pregnancies in 512 women. Numbers may not sum up to 700 due to missing data on maternal characteristics.

Year 2004-2008 was used as the reference group for all analyses.

β coefficients and 95% CIs were obtained for continuous outcome variables from linear regression models, and odds ratios (ORs) and 95% CIs were obtained for binary outcome variables from logistic regression models. P-values were calculated for trend.

^a^ Adjusted model 1: adjusted for age (years) and BMI (kg/m^2^).

^b^ Adjusted model 2: adjusted for age (years), BMI (kg/m^2^) and gestational weight gain (lbs).

^c^ We were able to extract neonatal glucose for the years 2008-2017 for infants who required stays in the neonatal intensive care unit (NICU). We discarded year 2008 as it only represents a small and not well-defined portion of the 2004-2008 period. The reference period for neonatal hypoglycemia is therefore 2009-2012. In addition, we excluded infants born in 2017 from the analysis due to a change neonatal hypoglycemia thresholds from 40 towards 45 mg/dL that year. P-values correspond to the comparison of 2013-2016 to 2009-2012.
