# Supplementary material for: Time trends in pregnancy-related outcomes among women with type 1 diabetes mellitus, 2004–2017
Source: J Perinatol. 2020 Jun 2;40(8):1145–53. doi: 10.1038/s41372-020-0698-x (PMC7375951; doi:10.1038/s41372-020-0698-x)
Supplement: Supplementary file 3 — Appendix [file 41372_2020_698_MOESM3_ESM.docx]

**Appendix: Missing data in the different time periods**

|  | **2004-2008 (n=214)** | **2009-2012 (n=215)** | **2013-2017 (n=271)** |
| --- | --- | --- | --- |
| **BMI** | 59 (27%) | 45 (20%) | 19 (7%) |
| **Age** | 1 (0.5%) | 14 (6%) | 6 (2%) |
| **ACR** | 28 (13%) | 46 (21%) | 29 (10%) |
| **HbA1c tri 1** | 21 (9%) | 25 (11%) | 18 (6%) |
| **HbA1c tri 2** | 8 (4%) | 15 (7%) | 17 (6%) |
| **HbA1c tri 3** | 11 (5%) | 25 (11%) | 16 (6%) |
| **Gestational weight gain** | 37 (17%) | 52 (24%) | 26 (9%) |
| **Birth weight** | 1 (0.5%) | 0 | 1 (0.4%) |
| **Gestational age at delivery** | 0 | 1 (0.5%) | 1 (0.4%) |
| **Mode of delivery** | 2 (0.9%) | 1 (0.5%) | 1 (0.4%) |
| **Neonatal hypoglycemia that required NICU** | 200 (93%) | 2 (0.9%) | 15 (5%) |
